# Building a Therapeutic Bridge Between Dogs and Humans: A Review of Potential Cross-Species Osteosarcoma Biomarkers

**DOI:** 10.3390/ijms26115152

**Published:** 2025-05-28

**Authors:** Agnieszka Dolnicka, Vibeke Fosse, Anna Raciborska, Agnieszka Śmieszek

**Affiliations:** 1 Laboratory of Preclinical Research and Cell Transplantation “In VetBio” at the Applied Biology Center, Department of Pharmacology and Toxicology, Faculty of Veterinary Medicine, Wroclaw University of Environmental and Life Sciences, Norwida 31, 50-375 Wroclaw, Poland; 2 Department of Clinical Science, University of Bergen, Jonas Lies vie 91B, 5021 Bergen, Norway; vibeke.fosse@uib.no; 3 Department of Oncology and Surgical Oncology for Children and Youth, Institute of Mother and Child, Kasprzaka 17a, 01-211 Warsaw, Poland; anna.raciborska@hoga.pl

**Keywords:** osteosarcoma, malignant bone tumor, translational biomarkers, comparative oncology, canine model, cross-species studies, one health

## Abstract

Osteosarcoma (OSA) is a naturally occurring malignant bone tumor in both humans and canines that is characterized by aggressive local behavior and a high propensity for metastasis. Despite advances in diagnostic methods and therapies, long-term survival rates have remained stagnant, underscoring the great need for the development of biomarkers serving in the prognosis and diagnosis of OSA across species. Biomarkers, molecular indicators of disease presence or progression, are pivotal tools in oncology, offering the potential to determine risk stratification, guide targeted therapies, and monitor treatment response. This review provides an in-depth analysis of the current landscape of OSA biomarkers, highlighting diagnostic and prognostic markers identified across species. We highlighted the role of biomarkers, including protein, cellular, metabolic, imaging, genetic, and epigenetic markers, in osteosarcoma diagnosis and prognosis and categorized them across multiple domains. Furthermore, this review explores the utility of the canine model in osteosarcoma research, emphasizing its relevance to human OSA due to comparable diagnostic approaches, prognostic indicators, and clinical manifestations. With this review, we aim to demonstrate that integrating biomarker research across species can deepen the understanding of osteosarcoma pathogenesis and advance knowledge of its underlying biology, ultimately paving the way for precision medicine strategies that benefit both human and veterinary oncology.

## 1. Introduction

Osteosarcoma (OSA) is the most common primary malignant bone tumor in both humans and dogs, with significant biological and clinical similarities between the two species [1,2]. Despite advancements in surgical techniques and adjuvant therapies, overall survival rates have remained relatively unchanged over the past decades [3]. The translational value of canine OSA as a spontaneous model for human disease has recently gained increasing recognition in comparative oncology [4]. Biomarkers offer a promising avenue for improving early diagnosis, prognostication, and therapeutic monitoring in OSA [5]. Biomarkers represent a key translational tool with the potential to enhance early detection, refine prognostic stratification, and guide therapeutic decision-making across species [4,5].

**Figure 1:**
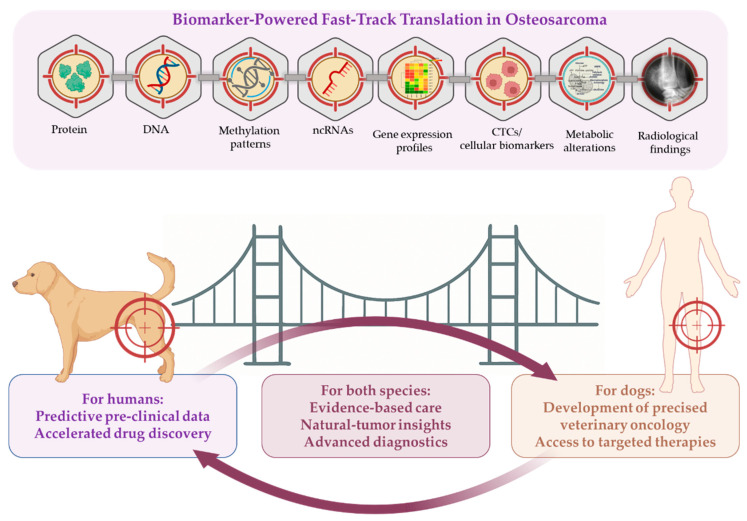
A visual summary of the review’s focus and aims. Conceptual framework highlighting the pivotal role of biomarkers development in accelerating translational progress in osteosarcoma, emphasizing improving diagnostic accuracy, monitoring disease progression, and guiding personalized therapeutic strategies across both human and canine models.

This review aims to critically evaluate current and emerging biomarkers in OSA and explore their potential to serve as a translational bridge between human and canine medicine (Figure 1).

The literature review was conducted through a comprehensive search of PubMed, Scopus, and Web of Science databases, targeting English language publications from 2000 to April 2025, with five articles older than the year 2000 due to the considerable impact of said articles. Keywords and keyword combinations such as “osteosarcoma”, “biomarkers”, “canine”, “human”, “comparative oncology”, “clinical features” “imaging diagnostics”, “parallels”, “plasma”, ”serum”, “tumor”, primary skeletal neoplasm”, and “X Rays” were employed to identify pertinent studies. Articles were included based on their relevance to biomarker discovery, validation, and translational potential due to shared molecular pathways, tumor biology, and clinical presentation between human and canine osteosarcoma.

## 2. Biomarker: A Concise Definition

A biomarker is a measurable indicator of a normal biological process, pathogenic condition, or response to a therapeutic intervention. The FDA-NIH (Food and Drug Administration – National Institutes of Health) Biomarker Working Group characterizes a biological marker as “a defined characteristic that is measured as an indicator of normal biological processes, pathogenic processes, or responses to an exposure or intervention, including therapeutic interventions” [6]. Biomarkers are essential in biomedical research and clinical practice, providing diagnostic, prognostic, predictive, pharmacodynamic, and monitoring information that facilitates disease assessment and therapeutic decision-making [7].

Biomarkers play a pivotal role in oncology by enabling early detection, precise diagnosis, and personalized therapeutic interventions, ultimately improving clinical outcomes. In osteosarcoma, they serve as critical tools for assessing tumor progression, metastatic potential, and treatment efficacy, allowing for a more tailored approach to disease management [8]. The discovery of reliable biomarkers facilitates non-invasive monitoring and allows for optimizing therapeutic strategies while minimizing unnecessary adverse effects [8,9]. Moreover, comparative biomarker research in human and canine osteosarcoma provides valuable insights into the underlying molecular mechanisms of the disease, fostering the development of novel targeted therapies [10]. Thus, this review will explore the current landscape of osteosarcoma biomarkers, emphasizing their translational significance and potential applications in both human and veterinary medicine.

The similarities in osteosarcoma’s clinical, genetic, and pathological features in canines and humans highlight the potential for using comparative oncology as a powerful tool in cancer research [11]. Canine osteosarcoma models can be utilized not only to understand better the molecular mechanisms driving tumor growth but also to develop novel therapeutic strategies that may benefit both species [4]. The study of naturally occurring osteosarcomas in dogs, alongside human clinical trials, offers a promising avenue for accelerating the discovery of more effective treatments and improving patient outcomes [12]. As the understanding of osteosarcoma continues to grow, the close collaboration between veterinary and human medicine will likely play a pivotal role in overcoming the challenges associated with this devastating disease [11].

## 3. Comparative Oncology: The Novel Approach to Biomarker Research

Comparative oncology studies naturally occurring cancers observed in companion animals, such as dogs, providing unique and powerful data to advance understanding and treatment of human cancer [10]. Observing and studying molecular, histopathological, and clinical features shared between human and canine malignancy is an approach that provides a biologically relevant platform that has the potential to surpass many limitations of traditional murine models [10,13,14]. Spontaneous tumor development in animals occurs within an immunocompetent host and under environmental exposures corresponding to those experienced by humans; these models share microenvironment interactions, metastatic patterns, and treatment challenges [15]. The overview of biomarkers, clinical advances, and translational implications in human and canine cancers is presented in Table 1.

Beyond OSA, comparative oncology between humans and canines has already demonstrated its utility in biomarker research in several tumor types including mammary tumors, lymphoma, and melanoma [16,17]. Examples of comparative oncology utility in canine mammary tumors research include the use of immunohistochemical subtyping based on HER2, ER, and PR status paralleled with classifications in human breast cancer; these findings suggest promising avenues in diagnostics and prognostic assessment for canines [18]. In lymphoma, shared expression patterns of CD20 and other B-cell markers have supported the use of spontaneous tumors in canines as preclinical models for non-Hodgkin lymphoma [19]. Current trials involving prodrug GS-9219, a nucleotide analogue 9-(2-phosphonylmethoxyethyl) guanine (PMEG) in canine non-Hodgkin lymphoma model, proved the anti-cancer properties of the tested substance on canines, providing possibly valuable information for human drug development [20].

In melanoma, particularly mucosal and acral subtypes, which are rare in humans but more common in dogs, genomic and immunologic analyses have contributed to the understanding of tumor etiology [21].

Creating and maintaining a collaborative infrastructure of institutions and networks facilitating cross-species oncology research, including the Comparative Oncology Trials Consortium (COTC) and academic centers engaged in One Health initiatives, poses a great opportunity for enabling integrated clinical trials, sharing biological samples and data, and accelerating the translation of novel cancer diagnostics and therapeutics between veterinary and human medicine [22].

All those successes underscore the translational power of comparative oncology, particularly for rare human cancers with limited patient population and limited treatment options. In addition, those examples highlight the potential of comparative oncology to support accelerated biomarker discovery and therapeutic innovations in osteosarcoma.

**Table 1:** Comparative overview of biomarkers, clinical advances, and translational implications in human and canine cancers. This table summarizes key biomarkers, clinical advances, and translational relevance across three major cancer types—mammary (breast), lymphoma, and melanoma—in both humans and dogs.

Cancer Type	Key Biomarkers	Clinical Advances in Humans	Clinical Advances in Dogs	Translational Impact	References
Mammary (Breast)	HER2, ER, PR, Ki-67, p53	HER2 and ER/PR are routinely used for prognostic assessment and to predict response to therapies	Similar expression of HER2 and ER/PR in canine mammary tumors (CMTs); under study for diagnostic and prognostic methods	Supports drug repurposing and predictive diagnostics across species	[18,23]
Lymphoma	CD20, CCL17 (TARC), PTPRK, Ki-67	Anti-CD20 antibody rituximab is added to standard therapy protocols to extend survival rates for B-cell lymphoma patients; GS-9219 not clinically tested yet	CD20-targeted therapies are under research in canine B-cell lymphoma; the prodrug GS-9219 is under research trials that demonstrated safety and efficacy for treatment of canine lymphoma	Shared immune markers enable parallel development of antibody therapies; use of spontaneous tumors in canines as preclinical models for non-Hodgkin lymphoma	[24,25]
Melanoma	BRAF, NRAS, PTPRK, PD-L1	BRAF mutations treated with vemurafenib; PD-L1 testing enables checkpoint inhibitor therapy	PD-L1 expressed in canine melanoma; c-KIT and TK inhibitors and other immunotherapies under clinical trials	Promotes checkpoint inhibitor research and immune profiling in veterinary oncology	[26,27]

While the rapid disease progression observed in canine models provides a distinct advantage for translational research and biomarkers discovery, facilitating the efficient evaluation of therapeutic efficacy and longitudinal disease outcomes, several limitations must be acknowledged to ensure comprehensive preclinical data interpretation. Canine models hold promise for the identification of translational biomarkers; however, differences in species-specific expression profiles and regulatory mechanisms may complicate the validation and clinical utility of such markers in humans.

Variations in age of disease onset, anatomical distribution, and responsiveness to combination therapies in osteosarcoma represent important factors that must be taken into account as a limitations due to the fact that those factors significantly influence disease modeling and therapeutic evaluation [13].

Moreover, interspecies divergences in immunocompetence, pharmacodynamic and pharmacokinetic parameters, as well as overarching systemic physiological processes, may substantially constrain the direct translatability of preclinical outcomes to human clinical paradigms, and vice versa, thereby limiting the bidirectional applicability of comparative findings [28]. In addition, the pronounced genetic polymorphism characteristics of the canine population can introduce substantial biological noise, thereby affecting statistical power and complicating data interpretation [29].

Furthermore, differences in environmental exposures and lifestyle factors, such as dietary patterns, physical activity levels, and even different access to healthcare interventions, may differentially modulate disease pathophysiology and trajectory, thus imposing additional limitations on cross-species extrapolation [30].

Finally, ethical considerations remain critical limitations in the use of companion animals in biomedical research, as such studies necessitate stringent adherence to regulatory frameworks, institutional oversight, and societal expectations regarding animal welfare and humane treatment [31,32].

## 4. Osteosarcoma: Shared Features Between Humans and Canines

Osteosarcoma (OSA) is the most prevalent primary malignant bone tumor in both humans and dogs [11], with canines serving as a valuable spontaneous model for disease investigation. Canine osteosarcoma provides a unique opportunity to advance human oncology through the field of comparative oncology, which aims to bridge the gap between human and veterinary medicine. This approach might be even more necessary, taking into consideration that OSA clinical outcomes in humans have not advanced considerably within the last 30 years [33]. Dogs are a good model for studying osteosarcoma because they develop the disease naturally, much like humans do, rather than through artificially induced tumors in laboratory animals. Furthermore, osteosarcoma is estimated to occur at least 10 times more frequently in dogs than in humans, making the canine population an important resource for studying the biology of the disease and testing new treatments [34]. Canines not only share comparable environmental exposures with humans, such as diet, lifestyle, and pollutant contact, but also exhibit significant similarities in genetic pathways, including those involved in disease development and immune response [13].

The bimodal age distribution is observed in both species; in humans, 70–75% cases of osteosarcoma occur in adolescents and young adults between the ages of 10 and 25, a period marked by rapid skeletal growth [35]. OSA accounts for 2.4% of all pediatric cancers [36]. A similar pattern is observed in dogs; the first, less pronounced peak occurs in juveniles under the age of three, accounting for about 6–8% of all cases. This early onset form of osteosarcoma may be linked to developmental processes, such as rapid bone growth during adolescence (puppyhood) [1]. This parallel suggests that specific biological processes related to growth and development may play a role in predisposing both species to the disease [13]. The second peak of osteosarcoma incidence is less distinct in humans; osteosarcoma can also occur in older adults between the ages of 60–70, although it is less common [37]. Osteosarcoma occurring in an older person used to be considered secondary due to underlying health conditions such as Paget’s disease or those who have undergone irradiation; however, it is now well documented that the second peak of the disease can occur de novo, without pre-existing bone disease [38] with some research suggesting that about half of the osteosarcoma cases in the elderly occurring de novo [39]. A more distinct second peak occurs in dogs, with most cases developing between the ages of seven and nine [40].

**Figure 2:**
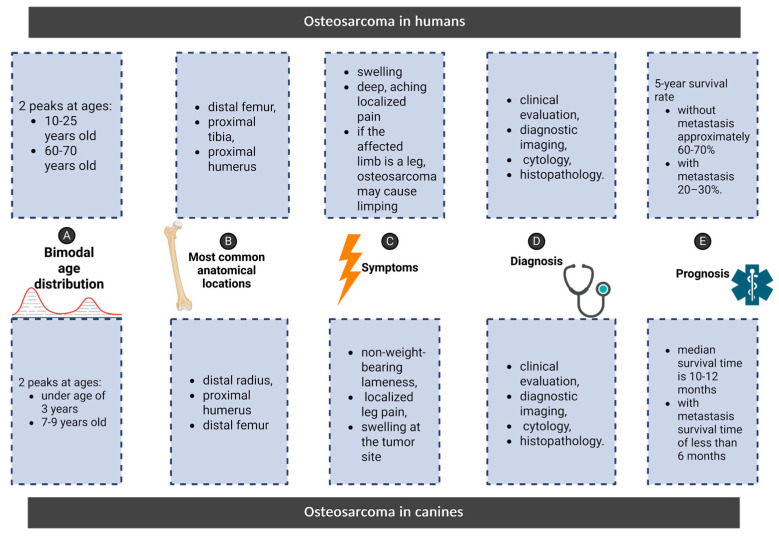
Comparison of osteosarcoma features in humans and canines including bimodal age distribution, most common locations, symptoms, diagnosis and prognosis.

Anatomically, the tumor affects similar locations in both species. In humans, the distal femur, proximal tibia, and proximal humerus are the most frequently affected bones, whereas in canines, the distal radius, proximal humerus, and distal femur are the most common sites of osteosarcoma. Furthermore, osteosarcoma in both humans and dogs primarily metastasizes to the lungs, followed by the bones and soft tissues [2,37]. Extraskeletal OSA (EsOSAs) has been reported in both humans and dogs. In both species, this location is rare, and in humans, it accounts for about 1% of all soft tissue sarcomas [41]. Human EsOSA cases are reported to be exclusively soft tissue sarcomas [42]. Meanwhile in dogs, EsOSAs can be divided into two subtypes: soft tissue sarcomas and mammary gland OSAs [43]. The summary of features characteristic for humans and dogs are also listed in Figure 2.

The tumor microenvironment, characterized by dynamic interactions between neoplastic cells and stromal components, including osteoblasts, osteoclasts, immune infiltrates, endothelial cells, and the extracellular matrix, is a common and critical feature of osteosarcoma biology in both dogs and humans [14]. The dynamic cellular interactions in the osteosarcoma microenvironment contribute to tumor growth, pathological angiogenesis, immune modulation, and the establishment of a pro-metastatic niche [14,44]. Notably, osteosarcoma demonstrates a strong tendency for pulmonary metastasis in both dogs and humans, with comparable molecular mechanisms and expression profiles implicated in pro-metastatic activity, including the upregulation of matrix metalloproteinases, vascular endothelial growth factor (VEGF) signaling pathways, and markers of epithelial-mesenchymal transition [14,45]. The involvement of VEGF-mediated angiogenesis and increased microvascular density in both species are linked to poorer clinical outcomes and heightened risk of pulmonary metastasis [14,46].

Both canine and human osteosarcomas exhibit immune infiltration in primary and metastatic lesions, particularly involving macrophages and lymphocytes, with shared expression of Th1-associated genes in T cells and comparable macrophage polarization patterns. Despite species-specific differences—such as a more distinct Th1 profile in humans and a stronger macrophage response in dogs—the underlying immunobiology shows sufficient overlap to support translational immunotherapy studies [14].

Moreover, undercharacterized components of the metastatic microenvironment such as type II pneumocytes (TIIPs) and cancer-associated fibroblasts (CAFs) represent important areas for future comparative research [14]. The contribution of endothelial cells to metastatic progression has been investigated in both human and canine osteosarcoma and is well established [47,48]. In turn, the specific roles of TIIPs and CAFs within the metastatic lung microenvironment remain poorly characterized in canine models. In human osteosarcoma, TIIPs have been implicated lung fibrosis, potentially contributing to tumor metastatic outgrowth. However, the extent to which these mechanisms are conserved in the canine lung remains unknown. Similarly, the role of CAFs in modulating the extracellular matrix, immune cell recruitment, and metastatic niche formation in dogs has yet to be thoroughly explored [14]. McGee et al. aptly indicated that the immunological characteristics of the tumor microenvironment play a critical role in osteosarcoma progression and treatment response, highlighting the need for further cross-species comparative studies. Such studies are essential to give opportunities for comparative research to uncover shared mechanisms related to osteosarcoma microenvironment regulation [13,14].

To summarize, uncovering shared cellular responses and therapeutic vulnerabilities in human and canine osteosarcoma is essential for advancing our understanding of the disease’s underlying biology. Such a comparative approach is crucial for enhancing diagnostic precision, identifying robust biomarkers, and guiding the development of more effective, translational treatment strategies for both species.

## 5. Parallels in Osteosarcoma: Comparing Clinical Features, Diagnosis, and Prognosis in Humans and Canines

### 5.1. Symptoms

In both humans and dogs, the clinical presentation of osteosarcoma shares remarkable similarities. In humans, the disease typically presents as swelling and deep, aching pain localized to the affected bone. About 80% of patients affected by the disease experience pain, most characteristically night pain [49]. If the affected limb is a leg, osteosarcoma may cause limping [37]. In dogs, the most common symptoms include acute non-weight-bearing lameness, localized leg pain, and swelling at the tumor site [2]. Upon physical examination, a painful, firm mass may be palpable. It is not uncommon for mild trauma to the bone to initially lead to a misdiagnosis of an orthopedic issue, such as a sprain or fracture. In some cases, affected animals may show temporary improvement with painkillers, further delaying accurate diagnosis. However, the recurrence of symptoms, particularly persistent pain and swelling, should prompt more comprehensive diagnostic testing, such as radiographs, biopsy, or advanced imaging. As in dogs, this pain is often mistaken for more benign conditions, such as growth plate issues or sports-related injuries, leading to delayed diagnosis and treatment. Given osteosarcoma’s aggressive nature and high metastatic potential, early recognition and intervention remain paramount in improving patient outcomes across species [1,2,50].

### 5.2. Diagnosis

In medicine, much like in veterinary medicine, the diagnostic process consists of a combination of clinical evaluation, diagnostic imaging, cytology, and histopathology. The initial step in the diagnostic process is usually diagnostic imaging. While the basic approaches to imaging in both humans and canines are analogous, a notable difference lies in the degree of specialization and technology available for humans compared to canines [41,50]. For instance, medicine utilizes a broader spectrum of targeted imaging techniques, including enhanced MRI protocols and innovative radiotracers for PET scans [51]. In contrast, canine imaging may often rely more heavily on standard radiographs and CT due to factors such as availability and cost. However, with the development of veterinary oncology, MRIs are more commonly incorporated in the OSA diagnostic process, particularly for cases requiring detailed soft tissue evaluation or precise surgical planning [50]. Radiographs are often a crucial step in veterinary medicine. The X-rays should be taken in two views, including cranio-caudal and lateral-medial, of the affected limb, including a joint above and beyond the affected bone [2]. The next step in the diagnostic process should consist of histopathological and immunohistochemical (vimentin) testing of surgically removed tumors, although fine needle biopsy is commonly used in veterinary medicine [52]. Differential diagnoses for humans include Ewing’s sarcoma, chondrosarcoma, and benign bone lesions such as osteomyelitis or fibrous dysplasia [53]. In dogs, differential diagnosis includes fungal or bacterial osteomyelitis, chondrosarcoma, and metastatic bone lesions, which are differentiated using histology, culture, or serological testing [54].

### 5.3. Prognosis

Prognosis when metastatic disease occurs is worse in both humans and dogs [37]. The 5-year survival rate for human osteosarcoma patients without metastasis is approximately 60–70%. In dogs, the disease progresses more rapidly due to their accelerated biology, allowing researchers to study treatment responses and disease outcomes over a shorter time. This makes canine osteosarcoma a valuable model for understanding the human condition and evaluating new therapies more efficiently. In metastatic cases, the prognosis declines markedly, with survival rates dwindling to approximately 20–30%. In canine standard treatment (amputation and chemotherapy), the median survival time is 10–12 months. Fewer than 20% of treated dogs survive beyond two years. Dogs with metastasis at diagnosis or in cases of inoperable tumors typically have a survival time of less than 6 months [1,2].

### 5.4. Biomarkers in Osteosarcoma: Translational Indicators of Disease and Therapeutic Response

**Figure 3:**
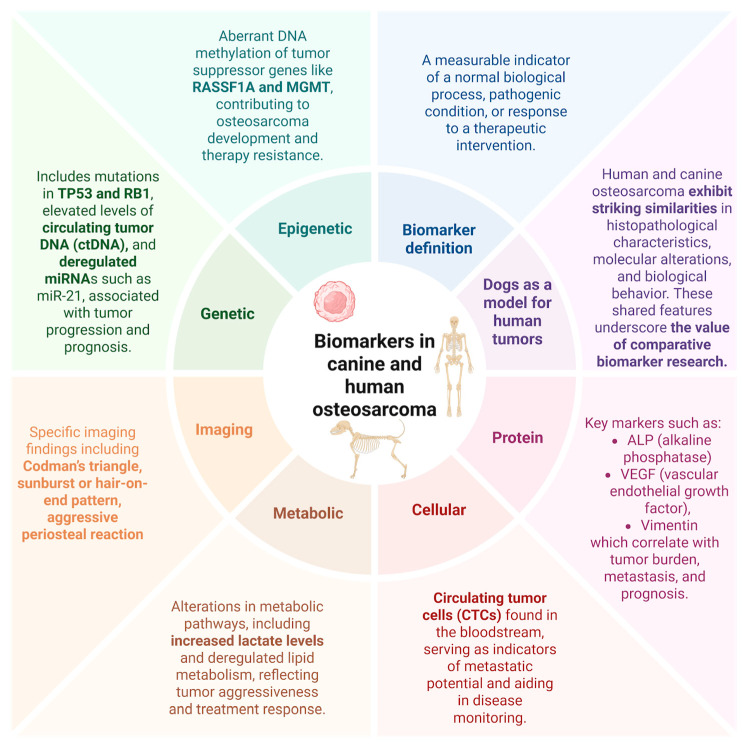
Categories of biomarkers based on an integrated network underlying osteosarcoma across species. The diagram shows diverse classes of biomarkers implicated in osteosarcoma pathogenesis, progression, and prognosis in both canines and humans.

Identifying osteosarcoma-specific biomarkers has significant clinical implications in precision medicine, guiding individualized treatment strategies. Comparative oncology provides a translational framework for biomarker discovery, facilitating the identification of common molecular pathways and advancing innovative therapeutic strategies applicable to both species (Figure 3).

## 6. Genetic and Epigenetic Biomarkers

The genetic characteristics of osteosarcoma are a complex topic that is still being studied. Even though the exact cause behind osteosarcoma development is not completely understood yet, researchers have identified several genetic mutations associated with the disease. Osteosarcomas tend to have a wide range of mutations and genetic instability, including a high frequency of somatic copy number alterations (SCNAs), chromosomal instability, aneuploidy, and chromothripsis, which is a phenomenon where massive chromosomal rearrangements occur in a single event. These mutations contribute to the tumor’s aggressive nature and resistance to treatment [1,36]. Key gene mutations that have been implicated in osteosarcoma appear to be similar between humans and canines [37,55]. Out of 56 identified recurrently amplified genes, 38 genes, such as *TFEB* and *MYC*, were found to be overlapping with pediatric OSA, while 69 genes, including *RB1* and *MSH3*, were recurrently deleted in both human and canine osteosarcomas [56].

*p53* is the most commonly mutated gene in both human and canine osteosarcoma. The incidence of p53 mutations varies between 15–42% in human and 24–47% in canine cases of OSA [57]. This gene is a well-known tumor suppressor that plays a critical role in cell cycle regulation, apoptosis, and DNA repair. Its mutations can often lead to a loss of tumor-suppressing functions. This allows cells to proliferate uncontrollably and contributes to the development and progression of tumors. Human studies have found a correlation between the upregulation of p53 and a decreased 3-year survival rate in osteosarcoma cases [58].

*RB1*, the retinoblastoma tumor suppressor gene, encodes retinoblastoma (pRB) protein. The RB family consists of 3 genes: *RB*, *p107 (RBL1),* and *p130 (RB2*). Mutations in this gene are known for their role in hereditary cancer syndromes. A mutation in *RB1* and its relatives significantly increases the risk of developing osteosarcoma, as it interferes with the cell’s ability to control the cell cycle, leading to unchecked cellular proliferation. In humans, RB gene mutations are associated with hereditary retinoblastoma [59].

The RUNX family of transcription factors plays a critical role in osteoblast differentiation from their progenitors of mesenchymal origin [60]. *RUNX2* is particularly important in regulating osteoblast maturation and maintaining proper cell cycle control, and it is described as a master regulator of osteogenic lineage commitment and differentiation. Disruption of *RUNX2* activity, especially through its overexpression, has been associated with impaired differentiation, increased proliferation, and other features characteristic of both in human and canine osteosarcoma [61,62]. Amplification of the *RUNX2* gene, often linked to chromosomal instability at 6p12-p21, appears to be an early event in osteosarcoma development and contributes to tumor progression [63].

Beyond its physiological role, increasing evidence implicates *RUNX2* in cancer biology by regulating tumor-related signaling pathways and downstream effectors. Collectively, these findings positioned *RUNX2* as a contributor to osteosarcoma pathogenesis and a potential biomarker for its monitoring. Although early data suggest that *RUNX2* expression may influence response to conventional chemotherapy, further clinical studies are needed to confirm its prognostic relevance. Given its central involvement in tumor progression, *RUNX2* is emerging as a promising therapeutic target, with ongoing research focused on elucidating its regulatory networks and developing RUNX-targeted treatment strategies in bone malignancies [60,63]. For example, the interaction between *RUNX2* and core binding factor beta (CBFβ) is a critical driver of malignancy in canine osteosarcoma, and disrupting this interaction using small-molecule allosteric inhibitors can suppress tumor cell growth, induce apoptosis, and enhance the effects of chemotherapy [64].

Osteosarcoma prevalence is also connected to mutations in other genes, such as phosphatase and tensin homolog deleted on chromosome ten (*PTEN*) and key components and regulators of the epithelial–mesenchymal transition (EMT) pathway [65,66]. *PTEN* is a negative regulator of the PI3K/Akt signaling pathway and a tumor suppressor gene. It encodes multiple proteins involved in regulating cell growth and differentiation and promoting apoptosis [67]. The expression rate of *PTEN* has been proven to be significantly lower in osteosarcoma human patient tissues than in adjacent cells [68,69]. Epithelial–mesenchymal transition (EMT) process plays a crucial role in osteosarcoma progression. Dysregulation of genes encoding key regulators of this pathway have also been implicated in metastasis development as they may enable tumor cells to acquire the ability to invade surrounding tissues and spread to distant sites, contributing to the high metastatic potential of osteosarcoma [70,71]. For example, *TGF-β* is a well-characterized and potent inducer of EMT in osteosarcoma cells, acting not only through the canonical Smad-dependent pathway but also via multiple non-canonical cascades—such as Ras/ERK, JNK/p38, PI3K/AKT, JAK/STAT3, and Rho GTPases—that collectively enhance the EMT process by promoting transcriptional reprogramming, cytoskeletal reorganization, and cell migration [72]. The process is associated with downregulating epithelial markers, such as E-cadherin, and the upregulating mesenchymal markers, including N-cadherin and vimentin, and thereby promoting phenotypic plasticity that results in enhanced cellular motility, invasiveness, and increased metastatic potential [73].

## 7. Small Non-Coding RNAs (microRNA/miRNAs)

MicroRNAs (miRNAs) are a class of small non-coding RNA molecules, typically 18–25 nucleotides in length, that play a crucial role in post-transcriptional gene regulation. They function by binding to complementary sequences in the 3′ untranslated region (3′ UTR) of target messenger RNAs (mRNAs), leading to translational repression or mRNA degradation. miRNAs are involved in the regulation of numerous biological processes, including cell proliferation, differentiation, apoptosis, and immune responses [74]. Onco-miRs, a subset of microRNAs involved in tumorigenesis, are considered valuable biomarkers since they exhibit tissue-specific and developmental stage-specific expression patterns, allowing for precise regulation of gene expression in this pathological context. Therefore, onco-miRs are not only potential diagnostic and prognostic biomarkers but also promising therapeutic targets in cancer management [75]. Numerous studies have investigated the role of miRNAs in human osteosarcoma (OS) by analyzing miRNA expression profiles, which have revealed distinct miRNA signatures associated with disease progression, metastatic potential, and therapeutic response [75,76,77].

In osteosarcoma and other cancers, specific onco-miRNAs, can be detected at altered levels in the serum, reflecting tumor presence, progression, or metastatic potential. Liquid biopsy approaches leveraging circulating miRNAs enable real-time disease monitoring, early detection of recurrence, and potentially guide personalized treatment strategies [78].

Dysregulation of specific miRNAs has been implicated in key oncogenic pathways, contributing to cell proliferation, apoptosis, invasion, and chemoresistance modulation. Despite significant advancements in understanding miRNA involvement in human OS, research on their role in canine OS remains comparatively limited. Nevertheless, existing studies on miRNA deregulation in naturally occurring canine malignancies, including spontaneous OS, suggest that aberrant miRNA expression similarly influences tumor biology in dogs [79]. Given the striking pathological and molecular similarities between human and canine OS, exploring miRNA dysregulation in the canine model holds substantial promise for identifying novel biomarkers and potential therapeutic targets applicable to both species [80]. Expanding research in this area may provide valuable insights into conserved oncogenic mechanisms and contribute to developing innovative miRNA-based diagnostic and therapeutic strategies.

MiR-214 plays a significant oncogenic role in osteosarcoma, promoting tumor progression, proliferation, migration, and invasion through multiple molecular pathways. It has been shown to regulate the Wnt/β-catenin [81] and MAPK signaling pathways [82], as well as directly target tumor suppressors such as CADM1 and TRAF3 [83], leading to enhanced metastatic potential. The consistent upregulation of miR-214 in human osteosarcoma tissues and cell lines highlights its potential as a diagnostic and prognostic biomarker, as well as a promising therapeutic target for osteosarcoma treatment. Given its strong association with osteosarcoma progression and metastatic potential, miR-214 emerges as a promising biomarker also in canine osteosarcoma. Ludwig et al. [84] showed that high expression of miR-214-3p was a strong prognostic biomarker in canine osteosarcoma, with multi-miRNA models further enhancing predictive accuracy for clinical outcomes. Its high expression consistently correlates with shorter overall survival and disease-free intervals.

MiR-21 is a key oncogenic microRNA with an essential role in the oncogenesis of various cancers, including human osteosarcoma. High levels of this molecule are related to enhanced tumor cell growth, invasion, and resistance to programmed cell death [77,85]. Its upregulation in osteosarcoma is associated with a worse prognosis, including higher tumor aggressiveness and lower survival rates. miR-21 directly targets tumor suppressor genes such as PTEN and PDCD4, activating pro-survival pathways, including PI3K/AKT signaling. Wang et al. also pointed out that miR-21, abundantly present in exosomes within the osteosarcoma tumor microenvironment, plays a crucial role in tumor progression, metastasis, angiogenesis, and immune evasion by influencing both cancer and surrounding cells [86]. Additionally, Li et al. [87] highlighted the potential role of miR-21 in evaluating chemotherapy response, distinguishing their study from previous research that primarily focused on miR-21 as a diagnostic and prognostic biomarker. Their findings indicated that miR-21 levels significantly differ before and after chemotherapy in patients who respond effectively to treatment. In this context, the study by Li et al. provides promising insights into the potential utility of miR-21 for monitoring therapeutic outcomes and guiding treatment adjustments [87].

In turn, the role of miR-21 in canine osteosarcoma remains unclear, with conflicting findings regarding its expression and function in tumor development. Our unpublished data indicate that miR-21 is elevated in the D17 osteosarcoma cell line, suggesting potential involvement in tumor progression. In contrast, Dailey et al. [80] used miR-21 as a reference gene, implying stable expression across their samples. These discrepancies may arise from differences in experimental models, sample types, or normalization strategies, highlighting the need for further investigation into the role of miR-21 in canine osteosarcoma. It is worth noting that the diagnostic potential of miR-21 is strongly recognized in canine mast cell tumors (MCTs), as it can distinguish between healthy and MCT-affected dogs, differentiate tumor origin (cutaneous vs. subcutaneous) and identify metastatic status, underscoring its value as a minimally invasive tool for detecting epigenetic alterations in canine tumors [88].

Another miRNA considered a valuable molecular biomarker is miR-223. Circulating miR-223 shows great potential as a non-invasive biomarker in liquid biopsy for osteosarcoma, with significantly reduced serum levels correlating with metastasis, advanced clinical stage, and poor prognosis. Dong et al. [89] showed high diagnostic accuracy of this miRNA (AUC 0.956), which highlights its utility for early detection and disease monitoring. Other findings supporting the role of miR-223-3p as a promising epigenetic regulator and prognostic indicator in osteosarcoma management also indicate its role in tumor progression. miR-223-3p directly targets CDH6 to inhibit tumor cell invasion, migration, and proliferation. Downregulation of miR-223-3p correlates with elevated catherin-6 (CDH6) expression and indicates poor prognosis and worse clinical outcomes, suggesting its potential as a liquid biopsy biomarker for predicting metastatic risk in osteosarcoma [90].

Based on current data in canine research, miR-223 appears to have a complex, context-dependent role in osteosarcoma, acting as both a tumor suppressor and a potential pro-metastatic factor. Tumor tissue expression may be elevated in aggressive cases of osteosarcoma dogs with shorter disease-free intervals (DFI), suggesting its involvement in tumor microenvironment modulation [80]. It was shown that miR-223 targets genes linked to cytoskeletal organization and cell adhesion, such as dystonin (DST) and catenin (cadherin-associated protein) alpha 2 (CTNNA2), which may promote invasion and metastasis when dysregulated. This duality highlights the complexity of miRNA-223 regulation in OS and the need for further research to clarify its role in disease progression [80].

Several tumor-suppressive miRNAs have also been identified in human and canine OS, including members of the let-7 family and miR-130a [80,91]. Those miRNAs are frequently downregulated in aggressive tumors. In addition, let-7b belongs to a well-characterized family of small non-coding RNAs, significantly regulating cell cycle dynamics. In osteosarcoma, let-7b directly targets and downregulates IGF1R, a key regulator of cell growth and invasion. The study by Zhang et al. showed that let-7b expression was notably reduced in osteosarcoma tissues and cell lines compared to normal controls. At the same time, IGF1R levels were elevated and inversely correlated with let-7b [91].

In turn, miR-130a levels significantly increase in osteosarcoma cells and tissues, and its elevated levels are associated with metastasis, poor prognosis, and aggressive clinical features [80]. MiR-130a plays a crucial role in promoting metastasis and the epithelial–mesenchymal transition in osteosarcoma by directly targeting and suppressing PTEN. In turn, decreased miR-130a expression was associated with an increased risk and a shorter disease-free interval in dog patients [92].

MicroRNAs are considered as tissue-specific biomarkers, as also noted in dog samples. Therefore, analyzing matched sample types is essential to differentiate between tumor-specific signals and those originating from the surrounding microenvironment or circulating cells. Such an approach is recommended as it ensures more accurate conclusions about disease mechanisms and progression. The study by Ludwig et al. underscores the fact that miRNA expression profiles differ significantly among tumor tissues, plasma, and cell lines derived from the same osteosarcoma patients. The sample type strongly influences the miRNA expression profile and affects its accurate interpretation and validation [93].

## 8. DNA Methylation Alterations

Comparative methylation profiling in human and canine osteosarcoma has revealed conserved DNA methylation clusters strongly associated with transcriptional programs governing cell proliferation and immune signatures, which are key determinants of tumor aggressiveness and prognosis. Noteworthy is the fact that global methylation patterns were found to reflect the underlying biology of the tumor itself, rather than being driven solely by the presence of immune cells, highlighting their value as markers of tumor behavior [94].

SETD2 is an enzyme responsible for trimethylation of histone H3 at lysine 36 (H3K36me3), which is a key epigenetic modification involved in gene expression and DNA repair. Although the tumor-suppressive function of SETD2 is well established in cancers such as renal cell carcinoma and leukemia, its role in osteosarcoma is still being uncovered [95,96]. Studies have linked SETD2 mutations to both human and canine OS, suggesting it may contribute to tumor development [36]. Mutations of SETD2 are less common in humans, occurring in 10% of cases of OSA [97]. Said mutations occur in approximately 21% of canine osteosarcoma cases [36]. The mutation of this gene has been linked to disruptions in genomic stability, further exacerbating the tumor’s aggressive nature [98]. SETD2 also interacts with and regulates TP53 [99], and co-occurring mutations in both genes have been observed in canine OS, implying a cooperative role in tumor progression [36]. The whole-genome sequencing of canine osteosarcoma samples performed by Gardner et al. [100] revealed recurrent alterations in the *SETD2* gene, including somatic point mutations, deletions, and chromosomal translocations in approximately 42% of cases. Additionally, a germline missense mutation in *SETD2* was identified in one case lacking somatic changes. Given the high amino acid conservation (91.8%) between human and canine SETD2, the observed mutations affect regions analogous to known mutation regions in human osteosarcoma [100].

## 9. Protein Biomarkers

The serum vascular endothelial growth factor (VEGF) family is a group of cytokines that serve as a key modulator of angiogenesis, the biological process responsible for the formation of new blood vessels that is crucial in numerous physiological mechanisms [101]. However, dysregulated VEGF expression is associated with various pathological conditions, notably oncogenesis, chronic inflammatory disorders, and ophthalmologic pathologies. Human and canine VEGF have similar amino acid sequences and are identical in the loop regions responsible for receptor binding [102]. Current research in humans agrees with VEGF blood and serum levels being associated with larger tumor sizes and a higher likelihood of metastasis in osteosarcoma human patients; however, this issue has been poorly investigated in canines [103]. The data on VEGF levels as a possible indicator of a response to chemotherapy are varied. One study found no connection between high levels of VEGF in human patients’ serum and response to chemotherapy [104], although varied chemotherapy responses, depending on VEGF levels, have also been reported when measuring pre- and post-chemotherapy levels of VEGF in humans [105]. In canines, research found a correlation between pre-treatment VEGF levels and overall survival, making VEGF a useful prognostic factor in canine OSA [103].

Alkaline phosphatase (ALP) is a hydrolase metalloenzyme found in all tissues but concentrated in bones, the placenta, liver, kidney, and intestines [106,107,108]. Alkaline phosphatase (ALP) comprises a family of four isoenzymes, each encoded by distinct genes and exhibiting tissue-specific expression, including hepatic ALP and bone-specific ALP (BALP). In clinical contexts, this enzymatic heterogeneity is critical, as total ALP (TALP) measurements reflect cumulative activity from multiple tissues, whereas BALP serves as a more selective biomarker for skeletal turnover and bone-related pathophysiology [107,109]. The enzyme plays a crucial role in skeletal activity, including processes like bone formation, mineralization, and metabolism [110]; therefore, it plays a significant role in the diagnostics and prognostics of bone pathologies, including osteosarcoma. ALP levels have been recognized as a prognostic factor in human osteosarcoma patients since 1959 [111]. In canine osteosarcoma research, the prognostic significance of ALP was reported as early as 1998 [112]. Pretreatment ALP levels in dogs are a prognostic indicator for OSA, and dogs with normal TALP (total alkaline phosphatase) and BALP (bone-related alkaline phosphatase) levels at the beginning of the therapy have longer median survival time than dogs with elevated TALP and BALP levels (12.5 months compared to 5.5 months) [113].

Dehydrogenase is an enzyme that transfers an anion of hydrogen from one molecule to another. Dehydrogenase lactate (LDH) plays a crucial role in converting lactate to pyruvate during glycolysis by providing NAD+ and converting it to NADH. Cancer cells tend to preferentially utilize anaerobic glycolysis even in the presence of oxygen, producing large amounts of lactate. This process has been known since around 1920s and is called the Warburg effect, making LDH an effective prognostic indicator and highlighting its utility in monitoring disease progression and response to therapy in both human and canine patients [1,114,115,116].

Vimentin (VIM) is a cytoskeleton building type III intermediate filament (IF) protein that is primarily expressed in mesenchymal cells and plays essential roles in maintaining cell integrity, shape, and motility [117]. Vimentin has the potential to be used as an epithelial-to-mesenchymal transition marker. EMT is a process occurring during both normal development and metastatic progression [118]; therefore, vimentin has the potential to be used both as a diagnostic factor, as well as a prognostic factor. Possible clinical applications of vimentin as a diagnostic tool in immunohistochemistry include the use of a combination of alkaline phosphatase staining with vimentin staining of lytic bone lesions in differentiating canine osteosarcoma from other bone neoplasms [61]. Using VIM as a prognostic factor improves when combined with other markers. The study by Sittiju et al. indicated that VIM expression significantly differentiated OSA patient samples from healthy donors and, when combined with ezrin and COL5A2, improved the model’s ability to predict disease presence and possible metastasis [119].

Similarly to humans, overexpression of vimentin in canine osteosarcoma is thought to contribute to enhanced tumor cell motility, invasiveness, and a more aggressive clinical behavior [120]. Moreover, Roy et al. reported that the metastatic canine OSA cell line HMPOS exhibited more than three times higher vimentin expression compared to the non-metastatic POS cell line [121]. In both human and canine osteosarcoma, elevated vimentin expression reflects the tumor’s mesenchymal origin and may further contribute to enhanced migratory and invasive behavior through EMT-like processes, underscoring its potential role in disease progression and metastasis [120,122,123]. However, despite its diagnostic relevance, VIM is also expressed in normal cells, particularly leukocytes, which may limit its specificity as a circulating biomarker unless combined with other OSA-specific genes for liquid biopsy applications [124,125].

## 10. Cell-Based Biomarkers

Circulating tumor cells (CTCs) originate from the primary tumor mass and enter the bloodstream through two main mechanisms—active intravasation or passive shedding. The detection and characterization of CTCs are valuable for understanding tumor progression and informing prognostic and therapeutic strategies [126]. CTCs detected in liquid biopsy can provide real-time insights into disease status and metastatic potential. In human oncology, the detection and quantification of CTCs are used as a minimally invasive tool to monitor disease progression and serve as a valuable prognostic indicator of patient survival [126,127]. Dai et al. described different CTCs subtypes, including epithelial (E-type), hybrid epithelial/mesenchymal (H-type), and mesenchymal (M-types), which may have different associations with osteosarcoma progression [128]. Notably, H-type and M-type CTCs, particularly those expressing IMP3, were more strongly linked to advanced disease stage and metastasis, suggesting their potential utility as specific biomarkers for metastatic risk in osteosarcoma. Indeed, the heterogeneity of the tumor and its biological diversity significantly influence CTC detection, emphasizing the importance of tailoring marker selection to the biological characteristics of each subtype since a universal approach may limit detection efficiency [126].

Even though CTCs can be detected in liquid biopsies of dogs with osteosarcoma [129], Wright et al. highlighted the challenges in recognizing circulating tumor cells as reliable biomarkers in dogs with osteosarcoma [130]. The CTCs were detected even after limb amputation and during chemotherapy, and their levels are often elevated prior to or alongside the detection of metastasis. Moreover, an increased number of CTCs were significantly associated with shorter survival times. However, no definitive CTC threshold could be established to predict impending metastasis, limiting its current clinical applicability. Differences in CTC dynamics between individual dogs and cell lines reflects the biological heterogeneity of the disease [129,130].

Taken together, current findings indicate that CTCs have potential as minimally invasive biomarkers for monitoring progression and metastatic risk in both human and canine osteosarcoma; however, their clinical application remains challenged by tumor heterogeneity, limited standardization of detection methods, and the need for subtype-specific approaches.

## 11. Radiological Findings in Translational Osteosarcoma Research

Radiological tests play a crucial role in the diagnosis and prognosis of osteosarcoma, both in humans and canines; these diagnostic tools enable early detection of the primary tumor, assessment of tumor extent, monitoring of therapeutic response, as well as early detection and monitoring of the metastatic disease. X-rays are a screening and initial assessment test in medicine [131], as well as in veterinary medicine [2]. Radiographic findings in osteosarcoma cases include expansile bone lesions, cortical defects, and specific findings called Codman triangle or a sun-burst reaction [132,133]. Differential radiological diagnosis in both species includes both benign and malignant conditions such as other primary bone tumors including chondrosarcoma, osteomyelitis, Langerhans cell histiocytosis, and lymphoma [132].

It is also worth mentioning that diagnostic imaging techniques, such as ultrasound, computed tomography (CT), and magnetic resonance imaging (MRI), play a crucial role not only in tumor detection and staging but also in guiding targeted sampling for histopathological evaluation [134,135]. By enabling precise localization of lesions, imaging facilitates the collection of representative tissue samples, thereby increasing the accuracy of histomorphology diagnosis, which is crucial for correct tumor characterization as it directly influences treatment decisions [135]. Integrating imaging with histopathology enhances diagnostic precision and clinical outcomes in osteosarcoma by enabling gaining more accurate samples for more accurate tumor characterization, improving staging accuracy, and guiding personalized treatment strategies [136]. Image-guided biopsies help minimizing sampling errors and reducing the risks of non-diagnostic or misleading histopathological findings, which is especially important in heterogeneous tumors like osteosarcoma [136,137]. In cases of anatomically challenging locations including the spine, pelvis, or areas adjacent to major blood vessels and nerves, imaging-guided biopsy sampling can help red reduce the risk of procedure-related complications [136,138].

Moreover, recently radiomics and radiogenomics has emerged as approaches that enhance traditional imaging by extracting quantitative features from scans and linking them with molecular tumor profiles. In osteosarcoma, these tools show potential for improving non-invasive diagnosis, risk stratification, and treatment planning. However, their clinical use remains limited due to the need for larger datasets, lack of standardized methodology, and validation of obtained biomarkers [136,139,140].

## 12. Conclusions

Osteosarcoma remains a highly aggressive malignancy in both humans and dogs, with limited progress in improving survival outcomes over recent decades. Biomarkers—ranging from genetic and epigenetic alterations to protein expression profiles, circulating tumor markers, and imaging-based indicators—offer critical insights into tumor behavior, prognosis, and therapeutic responsiveness. However, their clinical implementation remains limited due to tumor heterogeneity and a lack of robust validation. Comparative studies in human and canine osteosarcoma reveal striking biological similarities and support the dog as a valuable translational model for biomarker discovery. The identification of shared biomarkers, such as those involved in cell cycle regulation, immune infiltration, and epigenetic remodeling, may enable earlier diagnosis, more accurate risk stratification, and improved therapeutic targeting. Leveraging cross-species data not only deepens understanding of osteosarcoma pathogenesis but also holds the potential to accelerate the development of clinically relevant biomarkers and novel treatment strategies for both oncology and veterinary oncology.

## Abbreviations

CTCs	Circulating Tumor Cells
ctDNA	Circulating Tumor DNA
DST	Dystonin
EMT	Epithelial Mesenchymal Transition
LDH	Dehydrogenase Lactate
miRNA	MicroRNA
ncRNA	Non-coding RNA
OSA	Osteosarcoma
VEGF	Vascular Endothelial Growth Factor
VIM	Vimentin
